# Atypical atrial flutter ablation: clinical practice on patient selection, mapping, ablation strategies, and procedural endpoints—results from a European Heart Rhythm Association survey

**DOI:** 10.1093/europace/euaf307

**Published:** 2025-12-02

**Authors:** Giulio Falasconi, Antonio Berruezo, Martina Nesti, Maura Zylla, Mark T Mills, Michal Mazurek, Konstantinos Vlachos, Piotr Futyma, Martin Ruwald, Christian Heeger, Jarkko Karvonen, Laura Perrotta, Diego Penela, Julian Chun

**Affiliations:** Heart Institute, Teknon Medical Centre, Calle Villana 12, Barcelona 08022, Spain; Facultat de Medicina i Ciències de la Salut, Universitat de Barcelona (UB), c. Casanova, 143, Barcelona 08036, Spain; Heart Institute, Teknon Medical Centre, Calle Villana 12, Barcelona 08022, Spain; Cardiology Department, Fondazione Toscana Gabriele Monasterio, Pisa, Italy; Department of Cardiology, Heidelberg Centre of Heart Rhythm Disorders, Medical University Hospital, Heidelberg, Germany; Sheffield Teaching Hospitals NHS Foundation Trust, Sheffield, UK; Department of Cardiology, Congenital Heart Diseases and Electrotherapy, Silesian Centre for Heart Diseases, Skłodowskiej-Curie 9, Zabrze 41-800, Poland; Department of Cardiac Pacing and Electrophysiology, Hôpital Cardiologique du Haut-Lévêque, CHU de Bordeaux, Pessac, France; Medical College, University of Rzeszów, St. Joseph's Heart Rhythm Centre, Rzeszów, Poland; Heart Centre Capital Region, Copenhagen, Denmark; German Centre for Cardiovascular Research (DZHK), Partner Site Hamburg/Kiel/Lübeck, Lübeck, Germany; Department of Rhythmology, University Heart Centre Lübeck, University Hospital Schleswig-Holstein, Lübeck, Germany; Heart and Lung Centre, Helsinki University Hospital and University of Helsinki, Helsinki, Finland; Department of Cardiology, Arrhythmia Unit, Careggi University Hospital, Largo Brambilla 3, Florence 50134, Italy; Humanitas Research Hospital IRCCS, Rozzano, Milan, Italy; Department of Biomedical Sciences, Humanitas University, Pieve Emanuele, Milan, Italy; Cardioangiologisches Centrum Bethanien, Agaplesion Markus Krankenhaus, Frankfurt am Main, Germany

**Keywords:** Atypical atrial flutter, Survey, Catheter ablation

## Abstract

**Aims:**

Atypical atrial flutter (AAFl) encompasses a diverse group of macro-reentrant arrhythmias with variable circuits, presenting diagnostic and therapeutic challenges. This European Heart Rhythm Association (EHRA) survey aimed to assess current practices across European centres regarding the management of AAFl.

**Methods and results:**

A 26-item online questionnaire distributed by the EHRA Scientific Initiatives Committee yielded 214 responses from physicians in 36 countries. Catheter ablation was considered first-line therapy by 67.6% of respondents. In patients presenting in sinus rhythm with non-inducible clinical AAFl at the time of ablation, management strategies were heterogeneous, with combined pulmonary vein isolation and substrate ablation being the most common approach (46.8%). Activation mapping was the preferred method to define the circuit (63.7%), ahead of entrainment manoeuvers. Most respondents (87.1%) used ablation lines connecting scar or unexcitable tissue, whereas only 7.5% targeted the critical isthmus alone. The most frequent endpoints were validation of conduction block (73.1%), interruption of the clinical arrhythmia (71.0%), and non-inducibility of the clinical flutter (56.5%), while non-inducibility of any atrial flutter was rarely pursued. In patients without prior cardiac intervention, the left atrial anterior wall was perceived to be the most frequently involved structure (59.4%). Finally, in case of recurrence, 74.3% of respondents preferred redo ablation.

**Conclusion:**

This EHRA survey reveals consensus on ablation endpoints but marked variability in ablation timing and strategies when AAFl is non-inducible at the time of ablation, underscoring the need for standardized protocols and further collaborative research to optimize outcomes.

## Graphical Abstract

**Graphical Abstract:**
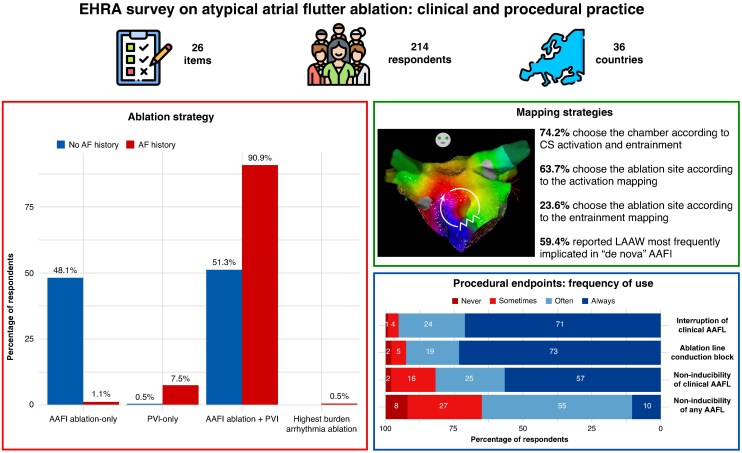

## Introduction

Atypical atrial flutter (AAFl) refers to macro-reentrant atrial tachycardias that are not dependent on the cavotricuspid isthmus (CTI), in contrast to the well-defined reentry circuit of typical CTI-dependent flutter.^1^ Atypical atrial flutter comprises a heterogeneous group of complex arrhythmias, often involving the left atrium (LA) and typically depending on scar-related circuits of diverse locations, most commonly following prior atrial fibrillation (AF) ablation or cardiac surgery.^2^ This marked heterogeneity translates into significant diagnostic and therapeutic challenges, as AAFl may present with diverse activation patterns and are frequently more difficult to characterize and treat than typical CTI-dependent flutter.^3^ Catheter ablation (CA) is an established treatment for AAFl.^4^ However, the optimal management approach is not yet standardized, and therapeutic outcomes remain heterogeneous. Published series have reported a wide range of acute success rates (∼73–100%) and recurrence probabilities at 1-year follow-up (roughly 7–53%).^5,6^ Moreover, several advanced mapping and ablation technologies are now available, which has led to considerable differences in how centres approach AAFl ablation.^6^ High-density three-dimensional electroanatomical mapping, the use of cardiac imaging, and the emerging ablation technologies, such as pulsed field ablation (PFA), can help delineate the arrhythmogenic substrate and the complex reentrant circuits and facilitate the identification and effective targeting of critical isthmuses.^7–10^ Despite these advances, AAFl remains challenging to map and ablate in many cases, due in part to its variable substrate location and complexity.

The aim of this European Heart Rhythm Association (EHRA) survey was to assess contemporary clinical practice in the management of AAFl. Specifically, the survey explored the indications and timing for CA, mapping techniques, procedural endpoints, and outcomes. The findings are expected to identify areas of consensus and divergence in current clinical practice and may help inform future recommendations and multicentre collaborative research.

## Methods

This physician-based survey was developed by the Scientific Initiatives Committee of the EHRA to investigate contemporary clinical practices regarding the management of AAFl across Europe. The questionnaire was developed by a panel of expert electrophysiologists, drawing upon recent scientific literature and collective clinical experience, with the aim of capturing real-world strategies, identifying current challenges, and informing future research directions.

The survey consisted of 26 questions divided into seven thematic domains: (i) participating centres’ data; (ii) baseline patient characteristics; (iii) treatment strategies; (iv) catheter ablation approaches; (v) mapping and ablation settings; (vi) anatomical substrate considerations related to flutter circuits; and (vii) procedural endpoints and post-ablation management. The questions included single- and multiple-choice formats, percentage estimates, ranking scales, and sliding bar inputs. The survey was administered through a secure online platform and distributed electronically through the EHRA Research Network, national electrophysiology societies, newsletters, and social media channels. This physician-based survey was open for 6 weeks beginning in May 2025 and was accessible to electrophysiology centres across Europe, irrespective of procedural volume or academic affiliation, to maximize a comprehensive and representative snapshot of current practice. Responses were collected anonymously and handled in accordance with applicable data protection regulations. An exploratory sub-analysis was performed by comparing responses from high- and low-volume ablation centres, according to their annual number of catheter ablation procedures (>500 vs. ≤500 ablations/year); for this analysis, *χ*^2^ or Fisher’s exact tests were used when appropriate, with *P* < 0.05 considered statistically significant. The findings are intended to support future EHRA recommendations and guide collaborative multicentre research aimed at improving the management of patients with AAFl. The full-survey questionnaire is available as Supplementary material online, *File S1*.

## Results

### Centre characteristics and baseline patient profile

The survey collected responses from 214 electrophysiologists across 36 countries. Most respondents reported working in medium-volume centres, with 40.5% indicating that their institution performs 150–500 ablation procedures annually. An additional 29.9% reported working in high-volume centres performing 501–1000 procedures per year, while 23.7% were based in very high-volume centres with more than 1000 annual ablations. At the individual operator level, 64.7% of respondents reported performing 1–50 AAFl ablations annually, and 21.9% reported 50–100 cases per year.

**Figure 1:**
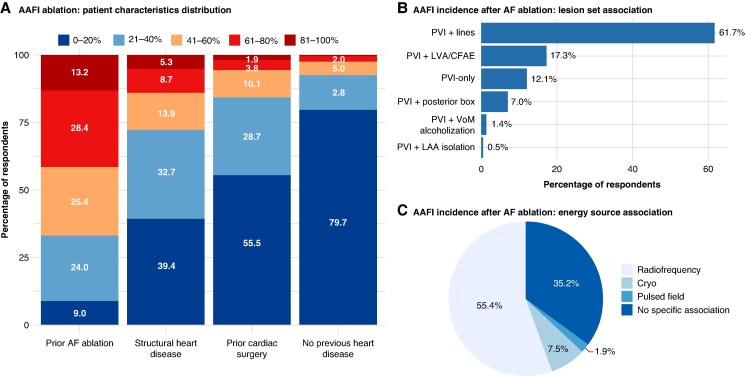
Clinical profile and procedural factors associated with AAFl occurrence. (*A*) Most respondents reported that AAFl patients frequently have structural heart disease, prior cardiac surgery, or previous AF ablation. (*B*) Combined PVI with additional linear lesions was perceived as the AF ablation strategy most commonly associated with later AAFl. (*C*) Radiofrequency energy was most often considered the modality linked to AAFl recurrence, reflecting its widespread use for linear lesion creation. AAFI, atypical atrial flutter; AF, atrial fibrillation; CFAE, complex fractionated atrial electrograms; LAA, left atrial appendage; LVA, low-voltage area; PVI, pulmonary vein isolation; VoM, vein of Marshall.

Most physicians (72.1%) estimated that structural heart disease was present in the range 0–40% of patients undergoing AAFl ablation. Similarly, prior cardiac surgery was reported in up to 40% of cases by 84.2% of respondents. In contrast, a history of previous AF ablation was commonly reported: the majority of participants (53.7%) indicated that 40–80% of their AAFl patients had undergone prior AF ablation. Finally, a substantial proportion (79.7%) estimated that <20% of patients had no previously diagnosed heart disease (*Figure 1A*).

When asked about AF ablation strategies most frequently associated with the development of subsequent AAFl, 61.7% of participants identified pulmonary vein isolation (PVI) combined with additional ablation lines (e.g. roof or mitral isthmus lines) as the most frequent one. Another 17.3% of respondents attributed AAFl recurrences to prior PVI procedures that included substrate modification targeting low-voltage areas or complex fractionated atrial electrograms (*Figure 1B*). Regarding energy sources, radiofrequency ablation was considered the modality most commonly associated with subsequent AAFl by 55.4% of respondents. In contrast, 35.2% reported no perceived association between the energy source used for AF ablation and the likelihood of developing AAFl (*Figure 1C*).

### Procedural planning across different clinical scenarios

**Figure 2:**
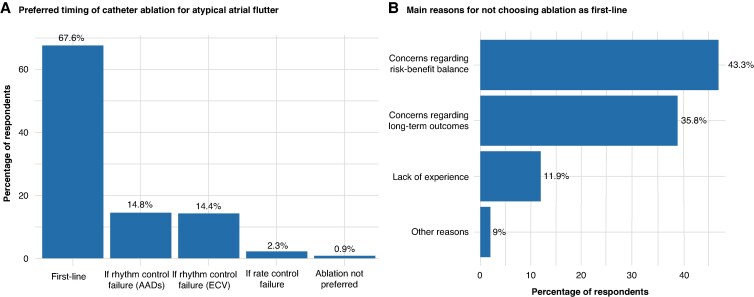
Preferred timing and barriers to first-line ablation in AAFl. (*A*) Most respondents favoured early CA as first-line therapy for AAFl. (*B*) Among those not using ablation as first-line strategy, concerns included the risk–benefit balance, uncertainty about long-term efficacy, and limited operator or institutional resources. AAD, antiarrhythmic drugs; AAFI, atypical atrial flutter; ECV, electrical cardioversion.

Regarding the timing of ablation for AAFl, the majority of physicians favour early intervention. Specifically, 67.6% of respondents reported performing CA as a first-line therapy, while 32.4% preferred a second-line approach after attempting alternative treatments. Among the latter group, 29.2% proceed to ablation only after the failure of a rhythm control strategy with antiarrhythmic drugs or electrical cardioversion, 2.3% reserve ablation for cases unresponsive to rate control therapy, and 0.9% prefer not to perform ablation for AAFl at all (*Figure 2A*). For those who do not routinely employ ablation as a first-line therapy, the survey explored the underlying reasons for this choice. The most cited concerns were the perceived risk-benefit ratio of the procedure (43.3%) and uncertainty regarding its long-term efficacy (35.8%). A smaller proportion (11.9%) attributed their reluctance to a lack of personal experience with AAFl ablation. A minority (9%) indicated other factors, such as limited institutional resources or excessively long waiting lists, which led them to prioritize other types of ablation procedures (*Figure 2B*).

At the time of scheduling ablation, the vast majority of physicians (89.3%) reported preferring that the patient remains in arrhythmia until the procedure.

**Figure 3:**
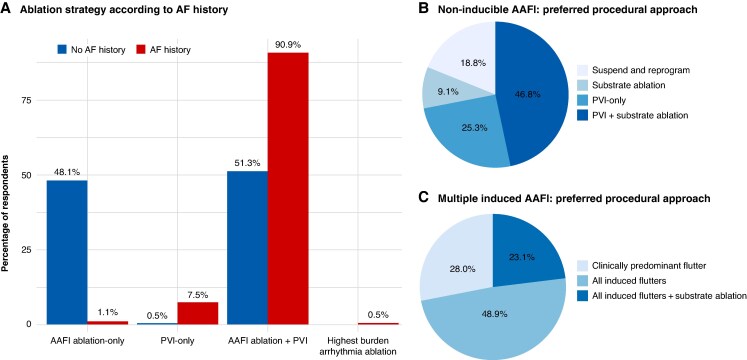
Procedural strategies for AAFl ablation according to presenting rhythm and concomitant arrhythmias. (*A*) In patients with concomitant AF, most respondents combined AAFl ablation with PVI, whereas in patients with a history of AF, strategies were more evenly split between AAFl-only ablation and AAFl ablation combined with PVI. (*B*) In patients in sinus rhythm with non-inducible AAFl, the most common approach was combined PVI and voltage-guided substrate ablation, followed by empirical PVI alone or rescheduling the procedure. (*C*) When multiple AAFl were inducible, many respondents targeted all flutters, while others limited ablation to the clinically predominant AAFl or added adjunctive substrate ablation. AAFI, atypical atrial flutter; AF, atrial fibrillation; PVI, pulmonary vein isolation.

**Figure 4:**
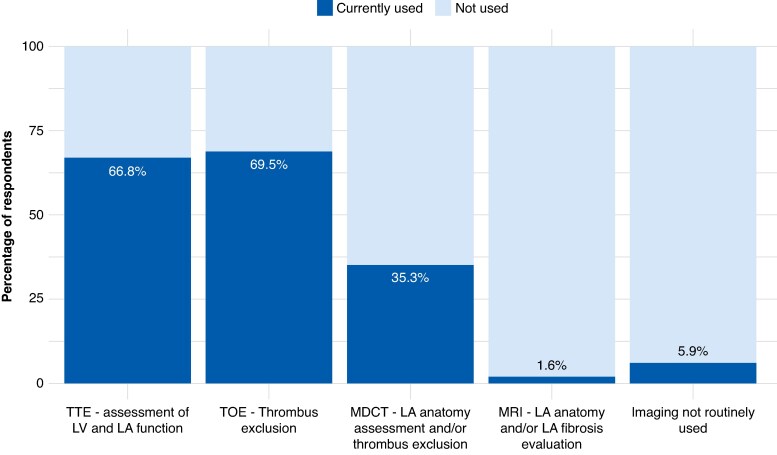
Pre-procedural cardiac imaging strategies in patients undergoing ablation for AAFl. Most respondents reported routine use of echocardiography (transoesophageal and transthoracic), whereas cardiac MDCT was used in roughly one-third of centres and late gadolinium-enhanced cardiac magnetic resonance was rarely employed. AAFI, atypical atrial flutter; LA, left atrium; LV, left ventricle; MDCT, multidetector computed tomography; MRI, magnetic resonance imaging; TOE, transoesophageal echocardiography; TTE, transthoracic echocardiography.

For patients presenting with isolated AAFl (no prior history of AF), 48.1% of respondents indicated that they perform AAFl ablation only, 51.3% reported empirically adding PVI even in the absence of documented AF, and 0.5% reported performing AF ablation-only without targeting the flutter. For patients with a history of both AF and AAFl, the vast majority of respondents (90.9%) stated they perform both ablation of the AAFl circuit and PVI as the initial ablation strategy; a minority of respondents reported performing PVI-only (7.5%), flutter ablation alone (1.1%), or tailoring the procedure to the arrhythmia with the highest clinical burden (0.5%) (*Figure 3A*). In patients presenting in sinus rhythm at the time of the procedure with a non-inducible AAFl, the most common strategy was to perform a combined PVI and a voltage map-guided substrate ablation, selected by 46.8% of respondents. An additional 25.3% opted for empirical PVI alone, while 18.8% reported suspending the procedure and rescheduling it for a time when the arrhythmia is present. A smaller proportion (9.1%) chose to perform substrate ablation based solely on voltage mapping without PVI (*Figure 3B*). When multiple AAFl were inducible during the same procedure, the majority of respondents (48.9%) reported targeting all inducible arrhythmias, without additional substrate modification, 28.0% reported a more selective approach, limiting ablation to the clinically predominant AAFl only, and a 23.1% indicated they ablate all induced flutters and also perform adjunctive substrate ablation (*Figure 3C*). Pre-procedural cardiac imaging was widely adopted among respondents, with most centres employing at least one modality before AAFl ablation: only 5.9% of physicians reported using no dedicated pre-procedural imaging modality beyond standard care. Transoesophageal echocardiography for thrombus exclusion (69.5%) and transthoracic echocardiography to assess left ventricular and atrial function (66.8%) were the most commonly used techniques. Cardiac multidetector computed tomography (MDCT) for LA anatomy evaluation and/or thrombus exclusion was used by 35.3% of respondents, while only 1.6% routinely performed late gadolinium-enhanced cardiac magnetic resonance imaging for the assessment of LA fibrosis (*Figure 4*).

### Mapping and ablation strategies and anatomical considerations

In the electrophysiology laboratory , regarding the initial mapping approach for AAFl, a clear majority (74.2%) reported selecting which atrium to map first based on the coronary sinus activation sequence and entrainment manoeuvers during the electrophysiological study. Less commonly, mapping was guided by patient history (8.6%), a stepwise approach starting from the right atrium (10.8%), or routine biatrial mapping (6.5%).

**Figure 5:**
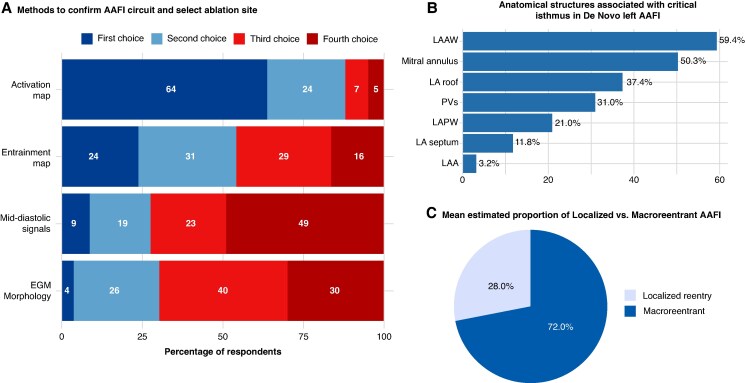
AAFl circuit mapping and anatomical considerations. (*A*) Activation mapping was ranked as the preferred method to confirm the circuit and select the first ablation site, whereas mid-diastolic signals and long or fractionated electrograms were least favoured. (*B*) In *de novo* left AAFl without prior cardiac interventions, the LAAW and mitral annulus were most frequently identified as part of the critical isthmus, followed by the LA roof and pulmonary veins. (*C*) Respondents estimated that macro-reentrant circuits account for the majority of AAFl cases, with localized reentry perceived as less common. AAFI, atypical atrial flutter; EGM, electrogram; LA, left atrium; LAA, left atrial appendage; LAAW, left atrial anterior wall; LAPW, left atrial posterior wall; PV, pulmonary vein.

When asked to rank the methods used to confirm the AAFl circuit location and select the initial ablation site, activation mapping emerged as the most preferred strategy, with 63.7% of respondents selecting it as their first choice and only 4.9% ranking it last. This was followed by entrainment manoeuvers, chosen as the first choice by 23.6% of respondents. In contrast, the detection of mid-diastolic signals between F waves was most frequently ranked last (48.9%), followed by electrogram morphology and fragmentation (29.7%) (*Figure 5A*). The vast majority of respondents (87.1%) reported preferring ablation lines connecting unexcitable tissue or scar areas as the lesion set of choice for treating macro-reentrant AAFl. Only a minority favoured single-point ablation on the critical isthmus (7.5%) or scar homogenization when the isthmus lies within a scarred region (5.4%).

Respondents were also asked to identify which anatomical structures are most frequently implicated as part of the critical isthmus in left AAFl circuits in patients without a history of prior cardiac interventions. The left atrial anterior wall (LAAW) was most commonly reported, being selected by 59.4% of respondents. The mitral annulus was also frequently cited (50.3%), followed by the left atrial roof (37.4%) and the pulmonary veins (31.0%) (*Figure 5B*). Macro-reentrant circuits account for the majority of AAFl encountered in clinical practice, with a mean estimated prevalence of 72 ± 16%. In contrast, micro-reentrant circuits were reported to represent ∼28 ± 16% of cases (*Figure 5C*).

The vast majority of respondents perceived biatrial AAFl as an infrequent arrhythmia. Specifically, 61.0% reported that both atria are involved in the tachycardia circuit in ≤5% of cases (13.9% indicating <1% and 47.1% estimating 1–5%). A further 28.3% of physicians estimated a prevalence between 5 and 10%, while only 10.7% reported biatrial flutters in more than 10% of cases. When asked about the clinical conditions most commonly associated with the development of biatrial AAFl, the majority of respondents (58.7%) identified previous cardiac surgery as the primary predisposing factor. Congenital heart disease was also frequently indicated (35.9%), followed by ablation involving the interatrial septum (34.2%), and LA extra-PVI ablation (29.3%). Interestingly, only 26.6% of participants attributed biatrial flutter to prior PVI alone, and an even smaller proportion (9.2%) to right atrial extra-PVI ablation. Very few respondents (3.3%) considered biatrial flutters to occur in the absence of any prior cardiac intervention. Finally, most respondents considered epicardial involvement in AAFl circuits to be relatively uncommon. Specifically, 64.1% estimated that epicardial bridging occurs in ≤10% of cases (26.1% reporting <5%, and 38.0% indicating 5–10%). An additional 25.5% believed that it occurs in 10–20% of patients, while only a minority (10.3%) reported higher prevalence rates exceeding 20%.

### Acute procedural endpoints and post-procedural management

When asked about the procedural endpoints routinely pursued at the end of AAFl ablation, nearly all respondents reported aiming to achieve interruption of the clinical arrhythmia during ablation (71.0% ‘always’, 24.2% ‘often’). Similarly, validation of conduction block across the ablation line was considered a primary endpoint, with 73.1% indicating they ‘always’ demonstrate it and 19.4% doing so ‘often’. Regarding non-inducibility of the clinical flutter, 56.5% of respondents reported pursuing it ‘always’, and 25.3% ‘often’. In contrast, non-inducibility of any atrial flutter was less frequently pursued as a consistent endpoint, with only 10.3% reporting ‘always’ aiming for it and the majority (54.6%) selecting ‘often’.

**Figure 6:**
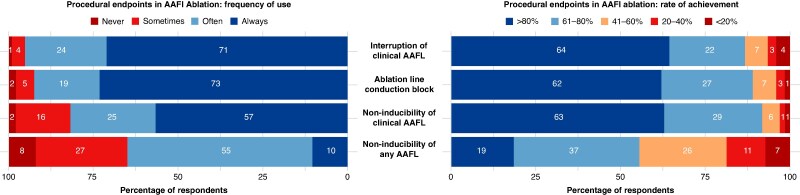
Procedural endpoints in AAFl ablation: reported frequency of use and achievement rates. Most respondents reported routinely pursuing interruption of the clinical arrhythmia, validation of conduction block, and non-inducibility of the clinical flutter, and achieving these endpoints in >80% of cases, whereas non-inducibility of any atrial flutter was targeted and obtained less frequently. AAFI, atypical atrial flutter.

Most respondents (>60%) reported achieving interruption of the clinical atrial flutter during ablation, conduction block across the ablation line, and non-inducibility of the clinical arrhythmia in over 80% of cases. In contrast, only 18.6% of respondents achieved non-inducibility of any atrial flutter in more than 80% of procedures. Most reported lower rates, with 37.1% indicating 60–80% and 25.7% indicating 40–60%. Regarding long-term outcomes, the estimated 1-year AAFl-free survival was reported to be >80% by 21.9% of respondents, whereas most participants (38.4%) estimated this rate between 40 and 60%, and 32.9% between 60 and 80%. Peri-procedural major complication rates were perceived as low, with 50% of respondents reporting a rate <1%, and another 46.1% indicating a rate between 1 and 5% (*Figure 6*).

The vast majority of respondents (78.6%) recommended short-term anticoagulation (2 months) followed by a long-term therapy based on the CHA_2_DS_2_-VA score. Only a minority favoured life-long anticoagulation irrespective of stroke risk (7.5%), long-term anticoagulation with possible suspension during follow-up (12.3%), or no anticoagulation at all (1.6%). The vast majority of respondents (74.3%) indicated redo CA as their preferred approach in case of arrhythmia recurrence. Other rhythm control strategies were selected by 19.3%, while ablate-and-pace (4.8%), rate control (1.1%), and no intervention (0.5%) were rarely chosen.

### Practice differences according to centre volume

In an exploratory analysis, we compared responses according to centre ablation volume (low-volume ≤500 vs. high-volume >500 procedures/year). High-volume centres more often reported combining AAFl ablation with PVI in patients without prior AF (61.2 vs. 40.0%; *P* = 0.010) and were more likely to perform combined PVI plus voltage map-derived substrate ablation in patients presenting in sinus rhythm with non-inducible AAFl (53.9 vs. 38.8%; *P* = 0.007), whereas low-volume centres more frequently chose to suspend the procedure and reschedule it (29.4 vs. 9.8%; *P* = 0.007). High-volume centres also reported more frequent use of cardiac MDCT for pre-procedural LA anatomical assessment (39.8 vs. 24.7%; *P* = 0.028), and a greater tendency to select the first atrium to map based on coronary sinus activation and entrainment manoeuvers (79.4 vs. 68.2%; *P* = 0.040), while low-volume centres more often chose to start with right atrial mapping (17.6 vs. 4.9%; *P* = 0.040). High-volume centres reported a greater likelihood of always validating conduction block across ablation lines (80.4 vs. 64.7%; *P* = 0.048), more frequently reported higher achievement rates of non-inducibility of any atrial flutter (*P* = 0.015), and more often perceived major complication rates <1% (62.2 vs. 32.3%; *P* = 0.013); in case of arrhythmia recurrence, they more frequently favoured redo ablation (82.5 vs. 64.7%; *P* = 0.008). Finally, high-volume centres were also more likely to attribute biatrial AAFl to prior left atrial extra-PVI ablation (40.6 vs. 15.5%; *P* < 0.001).

## Discussion

This survey provides valuable insights into current clinical practice for AAFl ablation across Europe. It captures a broad spectrum of clinical scenarios and decision-making pathways that are often underrepresented in the scientific literature, which tends to focus predominantly on AF ablation or reports from highly specialized centres. In contrast, the present data reflect the real-world experience of a wide range of institutions. Notably, more than 50% of respondents reported working in centres that perform over 500 ablation procedures annually, suggesting that the survey captures perspectives from both high- and medium-volume electrophysiology units. A graphical summary of the main survey findings is provided in the *Graphical Abstract*.

### Population characteristics

As reported in the literature, patients developing AAFl rarely have a completely normal cardiac history. Instead, most of them present with structural heart disease or have undergone prior cardiac interventions.^11–13^ Consistently, the majority of survey respondents estimated that AAFl patients commonly have a background of structural abnormalities or prior ablations. Furthermore, a substantial proportion of participants identified prior AF ablation—particularly when additional linear lesions beyond PVI were performed—as the most frequent predisposing factor for the development of AAFl during follow-up.^14^ Radiofrequency ablation was perceived as the energy source most commonly associated with subsequent AAFl, consistent with findings from previous studies. However, this perception may primarily reflect the widespread current use of radiofrequency energy, especially for developing ablation lines. As PFA becomes more widely adopted, this view may evolve. A contemporary state-of-the-art review has emphasized that PFA is a non-thermal, electroporation-based modality with relative myocardial selectivity and a favourable safety profile compared with thermal energies, particularly with respect to collateral oesophageal and phrenic nerve injury.^15^ Recent PFA series have started to characterize the burden of macro-reentrant atrial tachyarrhythmias after AF ablation. In a secondary analysis of the PULSED AF trial, Boersma *et al.*^16^ reported early recurrence of atrial tachyarrhythmias, including AAFl, in ∼30% of patients after PVI, with early recurrences strongly associated with late arrhythmia recurrence. Mills *et al.*^17^ described that fluoroscopy-guided PVI using a pentaspline-pulsed field catheter may result in incomplete PVI, inadvertent partial posterior wall isolation, and creation of macro-reentrant left atrial flutter circuits; the study showed that integration of the PFA catheter within a 3D electroanatomical mapping system can help identify gaps and avoid overlapping lesion sets. Together, these data suggest a non-negligible incidence of AAFl after AF ablation with pulsed field catheters, underlining the importance of lesion design and careful follow-up.

### Clinical scenarios and indications for ablation

The timing and indications for AAFl ablation remain heterogeneous across Europe, reflecting the complexity of clinical decision-making in this patient population. In the presented survey, 67.6% of respondents reported performing catheter ablation as a first-line treatment strategy. However, 32% preferred a delayed approach, typically after the failure of antiarrhythmic drugs or cardioversion. This variation likely reflects an ongoing risk-benefit consideration, especially given the procedural complexity and patient comorbidities often associated with AAFl. In this context, future studies aimed at better stratifying patients according to expected long-term benefit may help reduce this variability and support broader consensus on first-line ablation.

A particularly debated topic is the role of empirical PVI in patients with *de novo* AAFl and no prior history of AF. In the present survey, 48% of respondents reported performing PVI during the index AAFl ablation procedure, regardless of documented AF. This practice appears to be driven by the growing awareness that AAFl and AF often coexist or progress from one to the other, particularly in patients with atrial structural remodelling, and by the concern that AAFl may represent only one manifestation of an underlying atrial cardiomyopathy. Indeed, prior studies have shown that a substantial proportion of patients initially presenting with AAFl or typical flutter subsequently develop AF during follow-up, especially in the presence of left atrial dilation, low-voltage areas, or advanced age.^18^ From a clinical perspective, empirical PVI in this setting may therefore be viewed as an attempt to treat both arrhythmic manifestations of the underlying atrial structural disease, with the aim of reducing the future atrial tachyarrhythmia burden. However, this strategy remains controversial and there are no randomized trials specifically validating empirical PVI in patients with *de novo* AAFl and no documented AF. As a result, the incremental benefit of adding PVI must be balanced against the additional procedural time, complexity, and potential risks, and current evidence does not allow routine recommendation of this approach. Overall, the finding that almost half of respondents adopt empirical PVI in this scenario highlights both the perceived pathophysiological link between AF and AAFl and an important evidence gap that warrants dedicated prospective evaluation.

Similarly, in patients in sinus rhythm at the time of the procedure with non-inducible arrhythmia, 46.8% of respondents reported performing empirical ablation, most frequently combining PVI with voltage-guided substrate modification. This reflects a shift towards a more proactive and substrate-based approach, aimed at eliminating potential arrhythmogenic substrate even in the absence of a clearly inducible tachycardia. This trend is consistent with the emerging role of low-voltage area ablation in persistent AF ablation, where targeting atrial fibrosis or low-voltage zones has been associated with improved outcomes in selected populations.^19,20^ Given that the substrate plays a central role in sustaining macro-reentrant arrhythmias, this approach appears conceptually justified in AAFl patients as well. Nonetheless, nearly one-fifth of respondents (18.7%) preferred to reschedule the ablation for a time when the arrhythmia was present. This choice reflects the perceived procedural benefits of real-time mapping during tachycardia and the lack of definitive data supporting empirical substrate modification in the absence of inducibility.

These findings collectively underscore a pressing need for standardized protocols and prospective studies to clarify the optimal procedural strategy in specific clinical scenarios, particularly in patients without prior AF or with non-inducible arrhythmias at the time of ablation.

### Mapping strategies

The present survey highlights the use of activation mapping as the primary strategy for identifying AAFl circuits. Specifically, 63.7% of respondents ranked activation mapping as their first-choice method, and only 4.9% considered it the least useful, underscoring its important role in the electrophysiological definition of macro-reentrant arrhythmias. Entrainment manoeuvers also retained a relevant role, being selected as the preferred strategy by one quarter of respondents and as the second choice by a further third. Although entrainment mapping remains a robust mainstay, our survey suggests a shift in traditional practice,^21,22^ likely enabled by technological advances: activation mapping is now most commonly selected as the first-line strategy. In contrast, the identification of mid-diastolic signals and the use of long or fractionated electrograms were less commonly favoured, reflecting a general perception of their lower specificity or interpretative complexity. In summary, these results highlight a trend for growing reliance on mapping technologies over classical methods for circuit localization, especially as high-density electroanatomical mapping and sophisticated software processing tools become widely available.^23–25^

An additional point of interest is the identification of the LAAW as the most frequent location for the critical isthmus in *de novo* left AAFl (59.4%), followed by the mitral annulus (50.3%) and the LA roof (37.4%). This may be explained by recent findings in the literature: Pak *et al.*^26^ demonstrated the presence of low-voltage zones in the LAAW, specifically at the aorta–LA contact area. The exact mechanism of scar formation in this region remains unclear. However, LA remodelling and enlargement of the aortic root in patients with cardiovascular risk factors may contribute to the development of a low-voltage zone on the LAAW.^27^ These results emphasize the important role of detailed activation and substrate mapping in the LAAW in the case of AAFl occurrence in patients without prior cardiac interventions.

### Procedural endpoints and post-procedural management

There was a remarkable agreement among European electrophysiologists on the definition of procedural endpoints. The vast majority consistently pursue conduction block across ablation lines (73.1% ‘always’), interruption of the clinical arrhythmia during ablation (71.0%), and non-inducibility of the clinical flutter (56.5%). These are well-recognized criteria in the field and are considered surrogate markers for durable procedural success.^28^ However, non-inducibility of any atrial flutter was less frequently considered a routine endpoint (10.3% ‘always’). This likely reflects the practical limitations of prolonged stimulation protocols, including increased procedure duration, sedation time, and the risk of provoking non-sustained or unstable arrhythmias. In addition, the prognostic value of non-inducibility of non-clinical flutters remains uncertain, and aggressive attempts to eliminate every inducible circuit may lead to unnecessary ablation and further iatrogenic substrate modification. Notably, additional lesions—whether in the form of extensive linear ablation or wide areas of scar homogenization—may themselves create new lines of block and slow-conduction channels, potentially providing a substrate for novel organized atrial tachycardias. As a result, many operators appear to prioritize endpoints that are more clearly supported by clinical experience—such as interruption of the clinical arrhythmia, durable conduction block across the ablation line, and non-inducibility of the clinical flutter—while acknowledging that the role of ‘any-flutter’ non-inducibility as a formal endpoint still requires prospective validation.

Over 60% of respondents reported achieving the key procedural endpoints in more than 80% of cases, suggesting high confidence in acute success. Yet, reported 1-year arrhythmia-free survival rates remained modest, with most respondents estimating outcomes between 40 and 80%, confirming that medium-term success remains challenging, particularly in patients with extensive atrial disease or multiple arrhythmogenic substrates.^29^

Regarding post-ablation management, 78.6% of respondents reported using short-term anticoagulation followed by CHA_2_DS_2_-VA-guided continuation. Only 7.5% reported prescribing life-long anticoagulation regardless of stroke risk. This approach mirrors current guideline recommendations^30^ but reflects the lack of AAFl-specific anticoagulation trials. In case of arrhythmia recurrence, redo ablation was the strategy of choice for 74.3%, while rate control and ablate-and-pace strategies were rarely selected. These responses indicate a generally proactive approach and belief in the modifiability of the arrhythmic substrate with repeated interventions.

In this survey, high-volume centres tended to adopt more standardized and experience-driven workflows, which translated into a more intervention-oriented management strategy for atypical atrial flutter. These centres more frequently integrated PVI into their approach, employed substrate modification even in cases of non-inducibility, and relied more often on advanced imaging and structured electrophysiology manoeuvers during mapping. They also reported consistently validating conduction block, favouring redo ablation in case of recurrence, and perceiving lower complication rates. Collectively, these findings suggest that greater procedural exposure may lead to more consolidated procedural planning and greater confidence in achieving durable outcomes. Nonetheless, this exploratory comparison should be interpreted cautiously, as differences may reflect institutional organization, operator experience, and case mix rather than true disparities in efficacy or safety.

### Role of imaging and anatomical considerations

Pre-procedural imaging was widely adopted, with only 5.9% of respondents reporting no use of dedicated imaging. Transoesophageal echocardiography and transthoracic echocardiography were mostly used, while cardiac MDCT was utilized in approximately one-third of centres.^31^ Late gadolinium-enhanced cardiac magnetic resonance was rarely used, reflecting both limited availability and unclear added value in this population.^32,33^ These data highlight that, while pre-procedural multimodality cardiac imaging has seen increasing adoption in recent years for planning electrophysiology procedures,^34,35^ the choice of imaging modality is still largely centre-dependent and influenced more by resource availability than by evidence-based guidance.

Furthermore, this survey provides insights into the perceived prevalence and predisposing factors for biatrial AAFl. Only 10.7% of respondents reported encountering biatrial flutter in more than 10% of cases. Previous cardiac surgery (58.7%) and congenital heart disease (35.9%) were identified as the most relevant associated conditions, consistent with previous reports on scar-mediated macro-reentry in complex anatomical settings.^36^ Interestingly, only 3.3% believed biatrial circuits could occur without any prior cardiac intervention, reinforcing the notion that structural alteration is a key prerequisite for such complex arrhythmias.^37,38^

### Future directions

The findings of this survey highlight several unmet needs in the field of AAFl ablation. While certain procedural aspects—such as conduction block validation and the use of activation mapping as the primary mapping strategy—are managed with substantial consensus, considerable variability persists in other domains. These include the optimal timing of ablation, the role of empirical PVI in *de novo* AAFl patients without prior AF, and the procedural strategy in the absence of inducible arrhythmia. Future research should aim to determine the most appropriate timing and clinical management of AAFl, and to assess whether empirical approaches—such as PVI or substrate-guided ablation in non-inducible patients or those without prior AF—can improve long-term outcomes. Moreover, the potential role of novel technologies, including PFA, was not explored in this survey: implementation of PFA for AAFl ablation warrants dedicated investigation, particularly given recent concerns regarding the durability of single-shot PFA tools outside pulmonary veins and posterior wall.^39^

### Limitations

This survey-based study has several inherent limitations. First, as with all voluntary physician surveys, participation bias cannot be excluded. Respondents may not reflect the entire spectrum of European electrophysiology practices, and physicians with a particular interest or expertise in AAFl ablation may have been more likely to participate. Additionally, since data were collected at the individual level and not at the centre level, overlap between respondents from the same institution cannot be excluded. Second, although the survey achieved broad geographic coverage, the overall number of respondents may still be insufficient to fully capture the heterogeneity of practices across healthcare systems and procedural volumes. Third, all data are based on self-reported estimates rather than on audited procedural registries or prospectively collected information and are therefore subject to recall bias and inter-respondent variability. Reported figures for procedural success, arrhythmia-free survival, and complication rates should thus be interpreted as perceived operator estimates rather than as precise, adjudicated outcome measures. Finally, this survey provides a cross-sectional snapshot of practice patterns in May 2025. As highlighted in a recent state-of-the-art review on PFA,^15^ technologies, workflows and lesion sets are evolving rapidly, which may further modify the incidence and management of AAFl over time.

## Conclusions

This EHRA-based survey provides a comprehensive snapshot of current practices in AAFl ablation across Europe. The results reveal strong consensus in selected procedural approaches, such as using activation mapping as the primary mapping strategy and the routine validation of conduction block as a procedural endpoint. Conversely, significant heterogeneity persists regarding the timing of ablation, the use of empirical PVI and procedural strategies in patients without inducible arrhythmia at the time of ablation. These results underscore the need for standardized protocols regarding diagnostic evaluation, mapping techniques, and ablation strategies, which should be addressed through future consensus statements and multicentre clinical research collaborations.

## Supplementary Material

euaf307_Supplementary_Data

## Data Availability

The data that support the findings of this study are available from the corresponding author, upon reasonable request.
