# Supplementary material for: Atypical atrial flutter ablation: clinical practice on patient selection, mapping, ablation strategies, and procedural endpoints—results from a European Heart Rhythm Association survey
Source: Europace. 2025 Dec 2;27(12):euaf307. doi: 10.1093/europace/euaf307 (PMC12722029; doi:10.1093/europace/euaf307)
Supplement: euaf307_Supplementary_Data [file euaf307_supplementary_data.zip › Supplementary_file_1.docx]

Dear colleague,

The EHRA is conducting a survey on the management of patients undergoing atypical atrial flutter ablation in the daily clinical practice. Atypical atrial flutter is a type of supraventricular tachycardia characterized by a demonstrated reentrant circuit that does not involve the cavotricuspid isthmus.

The aims of this survey are:

- To inquire about the management of patients affected by atypical atrial flutter.
- To understand the current treatment and referral practices for atypical atrial flutter ablation.
- To obtain a comprehensive overview of the atypical atrial flutter ablation methodology employed across various EP laboratories.
- To identify possible improvements to the atypical atrial flutter ablation procedure in clinical practice.
- To identify the needs of the EP community for future research in this field.

The survey will take approximately 10 minutes of your time. Your answers will be considered anonymous. If you have any comments or questions about the survey, feel free to contact us at: antonio.berruezo@quironsalud.es

Thank you in advance for your contribution!

Antonio Berruezo, on behalf of the EHRA Scientific Initiatives Committee

**General aspects**

1. **In which country is your center located?**
2. **How many EP ablation procedures are performed at your center per ye­­­­ar?**

- Less than 150
- 150 to 500
- 500 to 1000
- More than 1000

1. **How many atypical atrial flutter ablations do you perform annually?**

- None
- 1 to 50
- 50 to 100
- More than 100

**Baseline characteristics**

1. **In patients undergoing atypical flutter ablation, what is the approximate distribution (in percentage) of those with the following characteristics?**

- Structural heart disease: ___%
- Previous cardiac surgery: ___%
- Previous AF ablation: ___%
- No previous diagnosed heart disease: ___%

1. **In your experience, which atrial fibrillation ablation approach is the most associated with recurrences manifesting as atypical atrial flutter?**

- PVI-only
- PVI + posterior box
- PVI + ablation lines
- PVI + low voltage areas / CFAE
- PVI + vein of Marshall alcoholization
- PVI + left atrial appendage isolation

1. **In your experience, which atrial fibrillation ablation energy source is the most associated with recurrences manifesting as atypical atrial flutter?**

- Radiofrequency
- Cryoablation
- Pulsed Field Ablation
- In my experience, there is no association with a specific energy source

**Aspects of atypical atrial flutter treatment**

1. **What is your preferred timing for catheter ablation of atypical atrial flutter?**

- First-line approach
- Second-line approach after failure of a rhythm control strategy with antiarrhythmic drugs.
- Second-line approach after failure of a rhythm control strategy with electrical cardioversion.
- Second-line approach after inadequate results of rate control strategy attempt
- I prefer not to perform ablation for atypical flutter

1. **What is the main reason ablation is not your procedure of choice in the management of atypical flutter? *(Answer only if the previous response is not "First-line approach")***

- Lack of experience
- Concerns about the risk/benefit ratio of the procedure

Concerns about the long-term outcomes of the procedure

- Other: please specify

1. **After scheduling an atypical atrial flutter ablation, do you prefer that the patient remains in atypical flutter?** *(To avoid the risk of non-inducibility and to target clinically significant flutter)*

- Yes
- No

**Ablation procedure approach**

1. **What is your ablation procedure approach for patients with atypical atrial flutter but no prior history of atrial fibrillation?**

- Atypical flutter ablation-only
- Pulmonary vein isolation-only
- Atypical flutter ablation + pulmonary vein isolation

1. **What is your preferred approach for patients with documented episodes of both atrial fibrillation and atypical atrial flutter?**

- Atypical flutter ablation-only
- Pulmonary vein isolation-only
- Atypical flutter ablation + pulmonary vein isolation
- Ablation of the arrhythmia with the highest burden

1. **What is your approach in patients who are in sinus rhythm at the time of the procedure and cannot be induced?**

- Suspend the ablation procedure and reprogram.
- Voltage map-derived substrate ablation
- PVI ablation – only
- PVI + voltage map-derived substrate ablation

1. **When inducing multiple atrial flutters, what ablation targets do you prefer during the procedure?**

- The clinically predominant atypical flutter
- All atypical flutters induced during the procedure
- All atypical flutters induced + substrate ablation

**Procedural setting**

1. **Which preprocedural cardiac imaging methodologies are routinely performed at your center prior to atypical atrial flutter? *(Multiple answers possible)***

- None
- Exclusion of atrial thrombus by transesophageal echocardiography
- LV and LA function assessment by transthoracic echocardiography
- PV and LA anatomy assessment by cardiac MDCT
- LA wall thickness assessment by cardiac MDCT
- LA fibrosis areas assessment by cardiac MRI

1. **Which initial approach to atypical flutter ablation do you prefer?**

- I choose the first atrium to map based on the patient's history
- I choose the first atrium to map based on the coronary sinus activation sequence and the EP study (entrainment maneuvers).
- I map the right atrium first and then map the left atrium only if passive activation is observed
- I routinely map both atria

1. **Which method do you rely on most to confirm the atypical flutter circuit and to select the first ablation point during ongoing flutter?** *(Rank in ascending order: 1 = not important, 4 = very important)*

- Entrainment maneuvers: ___
- Activation map (areas of slow conduction) : ___
- EGMs morphology, duration, and fragmentation: ___
- **Detection of mid-diastolic signals based on P waves during ongoing flutter: ___**

1. **Which lesion setting do you prefer for the ablation treatment of macro-reentrant atypical atrial flutters?**

- Ablation lines connecting unexcitable tissue or scar areas
- Single ablation points on the flutter critical isthmus
- Scar homogenization if the isthmus is located within a scar area

**Anatomical considerations**

1. **In your experience, which anatomical structures are most frequently associated with the critical isthmus of the circuit in atypical left atrial flutter in patients without a history of prior cardiac interventions? *(Multiple answers possible)***

- Left atrium anterior wall
- Left atrium posterior wall
- Left atrium roof
- Left atrium septum
- Pulmonary veins
- Mitral annulus
- Mitral isthmus
- Left atrial appendage

1. **In what percentage of patients undergoing atypical flutter ablation do you diagnose (sliding bar):**

- Microreentrant atypical atrial flutter: ….%
- Macroreentrant atypical atrial flutter:….%

1. **What percentage of cases do both atria form part of the tachycardia circuit, leading to a diagnosis of biatrial atypical flutter?**

- <1%
- 1-5%
- 5-10%
- 10-20%
- >20

1. **Which conditions do you consider to be most commonly associated with the onset of biatrial atypical flutters *(Multiple answers possible)*?**

- Previous cardiac surgery
- Previous pulmonary vein isolation
- Ablation with interatrial septum extra-pulmonary vein lesions
- Ablation with right atrial extra-pulmonary vein lesions
- Ablation with left atrial extra-pulmonary vein lesions
- Congenital heart disease
- No prior cardiac interventions

1. **In your experience, in what percentage of cases do epicardial structures form part of the critical tachycardia circuit (epicardial bridging)?**

- <5%
- 5-10%
- 10-20%
- 20-40%
- >40%

**Procedural endpoints and post-procedural aspects**

1. **Which procedural endpoint do you aim to demonstrate at the end of each procedure? (Select one option for each endpoint)**

- Interruption of clinical atrial flutter during ablation (always/ most / sometimes/ never)
- Validation of conduction block across the ablation line (always/ most / sometimes/ never)
- Non-inducibility of the clinical atrial flutter (always/ most / sometimes/ never)
- Non-inducibility of any atrial flutter (always/ most / sometimes/ never)

1. **At the end of your ablation, what are the percentages for the following outcomes of your procedures *(put NA if the endpoint is not used)*:**

- Interruption of clinical atrial flutter during ablation: ___%
- Conduction block across the ablation line: ___%
- Non-inducibility of the clinical atrial flutter: ___%
- Non-inducibility of any atrial flutter: ___%
- Peri-procedural major complication rate *(treatment-related adverse event requiring further therapy, increased level of care, or prolonged hospitalization)*: ___%
- 1-year atypical atrial flutter-free survival rate: ___%

1. **What is your post-ablation anticoagulation recommendation after a successful atypical flutter ablation, in patients without history of atrial fibrillation?**

- I do not recommend anticoagulation after a successful atypical flutter ablation
- Short-term anticoagulation (2 months), and successive long-term anticoagulation according to the CHA2DS2-VA score
- Life-long anticoagulation regardless CHA2DS2-VA score
- Long-term anticoagulation, with suspension if atrial arrhythmia-free during the follow-up

1. **What is your preferred approach in case of arrhythmia recurrence?**

- None
- Rhythm control (electrical cardioversion and drug therapy)
- Rate control (drug therapy)
- Redo catheter ablation
- Ablate and pace
