# Supplementary figures and images for: Atypical atrial flutter ablation: clinical practice on patient selection, mapping, ablation strategies, and procedural endpoints—results from a European Heart Rhythm Association survey
Source: Europace. 2025 Dec 2;27(12):euaf307. doi: 10.1093/europace/euaf307 (PMC12722029; doi:10.1093/europace/euaf307)

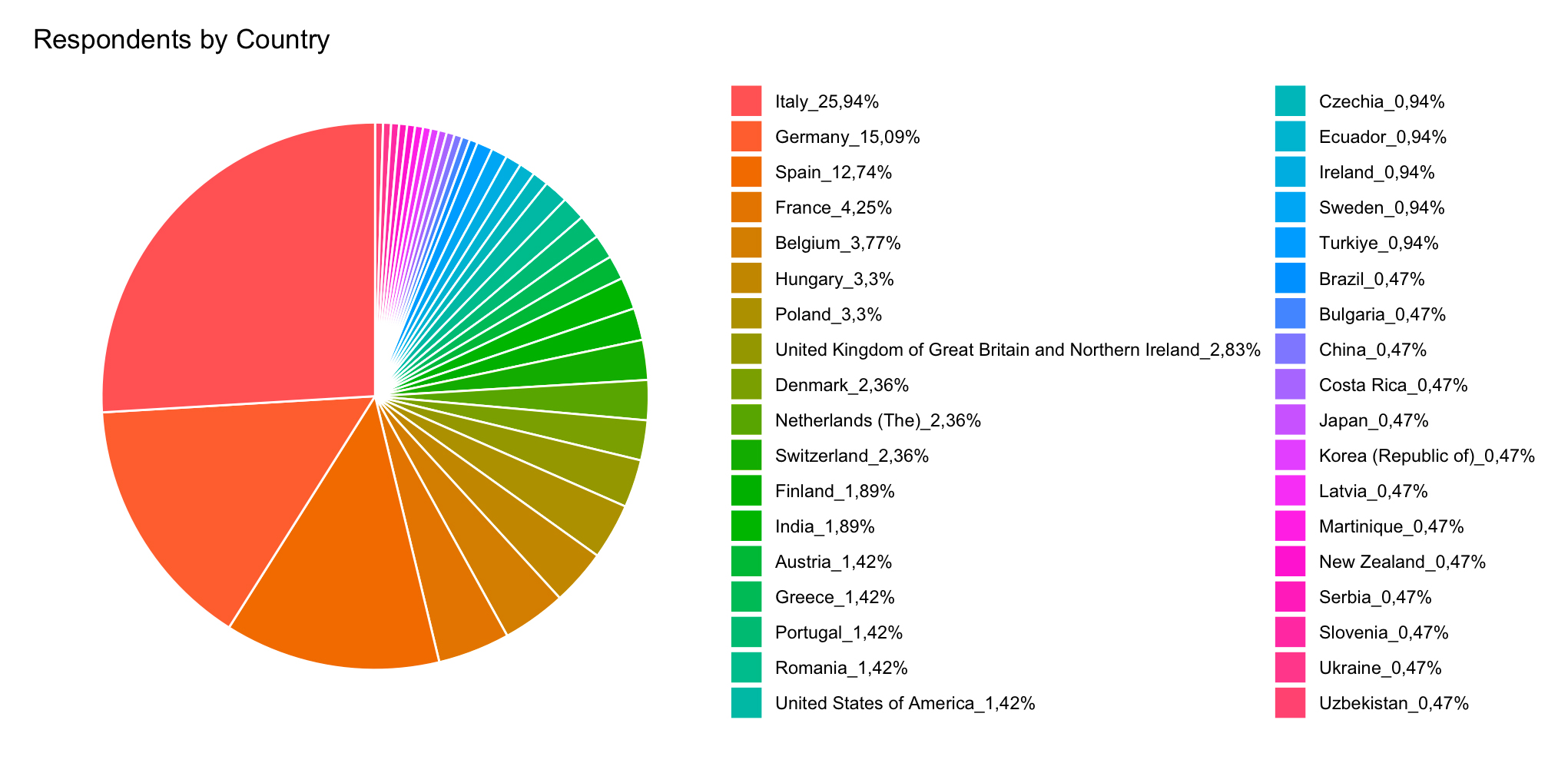

Supplement: euaf307_Supplementary_Data [file euaf307_supplementary_data.zip › Supplementary_figure_1.jpg]

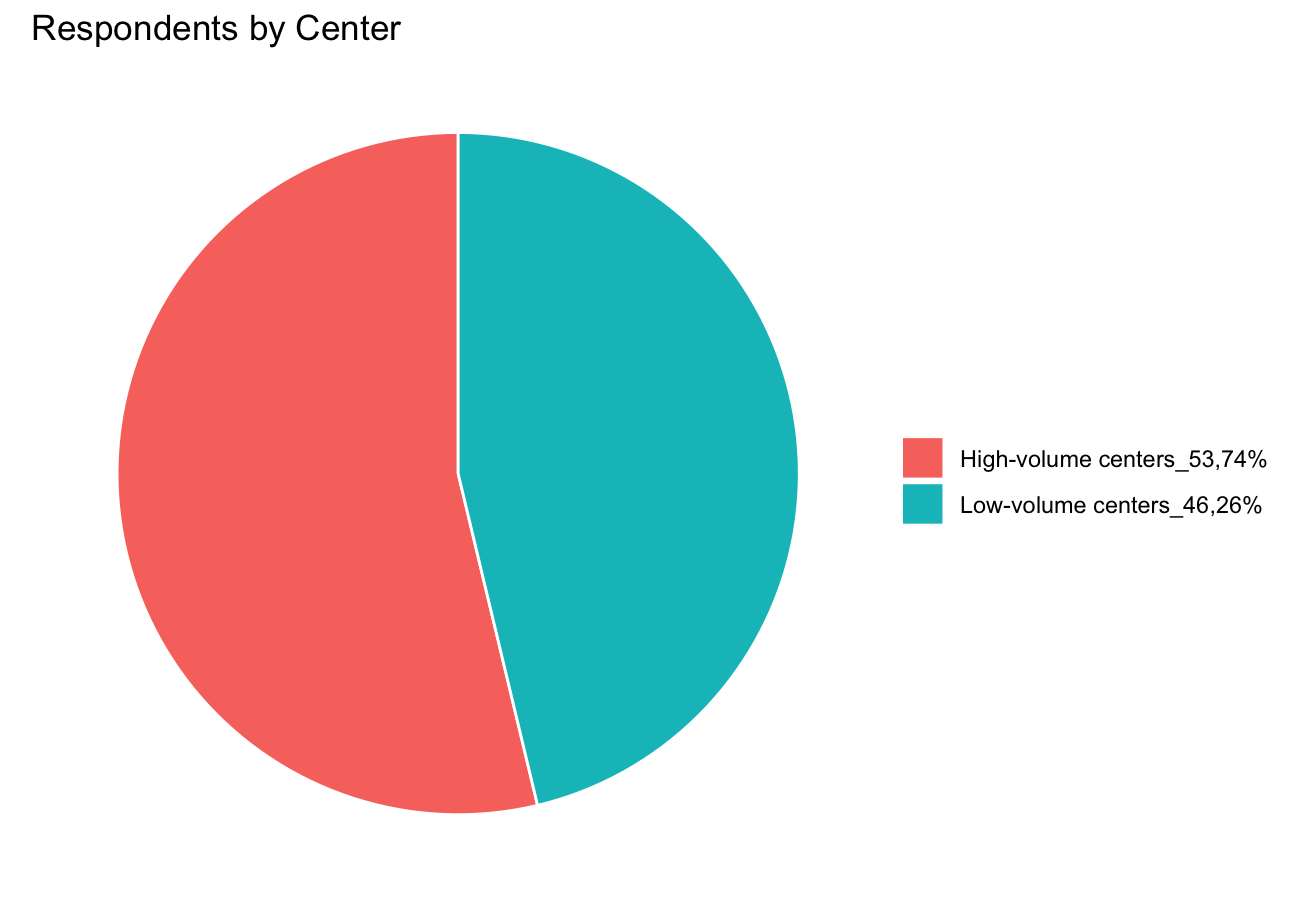

Supplement: euaf307_Supplementary_Data [file euaf307_supplementary_data.zip › Supplementary_figure_2.jpg]
